# Characterization of Awp14, A Novel Cluster III Adhesin Identified in a High Biofilm-Forming *Candida glabrata* Isolate

**DOI:** 10.3389/fcimb.2021.790465

**Published:** 2021-11-15

**Authors:** Jordan Fernández-Pereira, María Alvarado, Emilia Gómez-Molero, Henk L. Dekker, María Teresa Blázquez-Muñoz, Elena Eraso, Oliver Bader, Piet W. J. de Groot

**Affiliations:** ^1^ Albacete Regional Center for Biomedical Research, Castilla - La Mancha Science & Technology Park, University of Castilla-La Mancha, Albacete, Spain; ^2^ Institute for Medical Microbiology, University Medical Center Göttingen, Göttingen, Germany; ^3^ Mass Spectrometry of Biomolecules, Swammerdam Institute for Life Sciences Amsterdam, University of Amsterdam, Amsterdam, Netherlands; ^4^ Department of Immunology, Microbiology and Parasitology, Faculty of Medicine and Nursing, University of the Basque Country (UPV/EHU), Bilbao, Spain

**Keywords:** adhesion, GPI protein, cell wall protein, host-pathogen interactions, *Candida glabrata*, candidiasis, biofilm

## Abstract

*Candida glabrata* is among the most prevalent causes of candidiasis. Unlike *Candida albicans*, it is not capable of changing morphology between yeast and hyphal forms but instead has developed other virulence factors. An important feature is its unprecedented large repertoire of predicted cell wall adhesins, which are thought to enable adherence to a variety of surfaces under different conditions. Here, we analyzed the wall proteome of PEU1221, a high biofilm-forming clinical strain isolated from an infected central venous catheter, under biofilm-forming conditions. This isolate shows increased incorporation of putative adhesins, including eight proteins that were not detected in walls of reference strain ATCC 2001, and of which Epa22, Awp14, and Awp2e were identified for the first time. The proteomics data suggest that cluster III adhesin Awp14 is relatively abundant in PEU1221. Phenotypic studies with *awp14Δ* deletion mutants showed that Awp14 is not responsible for the high biofilm formation of PEU1221 onto polystyrene. However, *awp14Δ* mutant cells in PEU1221 background showed a slightly diminished binding to chitin and seemed to sediment slightly slower than the parental strain suggesting implication in fungal cell-cell interactions. By structural modeling, we further demonstrate similarity between the ligand-binding domains of cluster III adhesin Awp14 and those of cluster V and VI adhesins. In conclusion, our work confirms the increased incorporation of putative adhesins, such as Awp14, in high biofilm-forming isolates, and contributes to decipher the precise role of these proteins in the establishment of *C. glabrata* infections.

## Introduction

*Candida glabrata* is the second or third most common cause of nosocomial candidiasis (Pfaller et al., 2010; Pfaller et al., 2014), most frequently affecting elderly and neutropenic patients receiving fluconazole prophylaxis (Bodey et al., 2002; Malani et al., 2011). Its high mortality rate and its frequent resistance to antifungals make it a pathogen of clinical importance and, therefore, it is essential to know its virulence factors (Lamoth et al., 2018). *C. glabrata* does not undergo yeast-to-hypha transition as *Candida albicans* does, yet it has developed other virulence factors such as the production of hydrolytic enzymes (e.g., phospholipases, proteases, and hemolysins), the ability to evade host defenses, tolerance to high-stress environments, and resistance to neutrophil killing (Silva et al., 2012; Hassan et al., 2021). Furthermore, it contains an extraordinarily large repertoire of genes encoding putative GPI-modified cell wall adhesins in its genome conferring the yeast high adhesion and biofilm formation capacity onto host epithelia and abiotic surfaces (De Groot et al., 2013; Xu et al., 2020).

The cell wall of *Candida* plays a crucial role in its pathogenesis. The structure of the cell wall can generally be divided into two layers. An inner layer contains the polysaccharides β-1,3-glucan, β-1,6-glucan and chitin, whereas the outer layer of the cell wall is mostly composed of highly glycosylated glycophosphatidylinositol-modified cell wall proteins (GPI-CWPs) whose C-terminal ends are covalently linked to β-1,6-glucan through a remnant of their GPI anchor (Klis et al., 2009). Functions of the GPI wall proteins are manifold, for instance, some are involved in cell wall synthesis and remodeling while others have protective or structural functions, protease activity, or play a role in cell adhesion (De Groot et al., 2005). Fungal adhesins are large, modular proteins with large C-terminal domains of low complexity, which probably act as stalks and emanate through the cell wall to present N-terminal ligand-binding domains at the outermost cell surface to exert their adhesive function (De Groot et al., 2013).

Well-studied adhesins in *C. glabrata* are the Epa proteins, a family of about 20 proteins that have been shown to play a role in adhesion to epithelial cells and to bind terminal galactosyl residues onto host glycoproteins (Zupancic et al., 2008; Hoffmann et al., 2020; Xu et al., 2020). However, detailed genomic analyses have shown that the genome of *C. glabrata* contains about 70 putative GPI adhesin-encoding genes (De Groot et al., 2013; Xu et al., 2020). Most of these are localized close to telomeres where subtelomeric silencing may provide tight regulation in response to different environmental (host) conditions (De Las Peñas et al., 2015). Phylogenetic analysis of their N-terminal ligand-binding domains divided the putative adhesins into seven clusters with low levels of sequence similarity between these clusters, raising questions about the functionality and binding ligands of the thus far unstudied adhesins. On the other hand, the C-terminal domains of about half of the putative adhesins contain large internal repeats that are shared by proteins across the different adhesin clusters, demonstrating clear functional relationships between these proteins (De Groot et al., 2008; De Groot et al., 2013).

Proteomic analysis has shown that the *C. glabrata* CWPs identified by tandem mass spectrometry can be divided into two groups: (i) a stable core proteome, including carbohydrate-active enzymes, putative non-enzymatic glucan crosslinkers (Cwp1 and Pir families), and Ecm33 family members, among others, and (ii) a variable set of putative adhesins (Gómez-Molero et al., 2015). While the core proteome hardly shows any changes between different strains or growth conditions, clear strain-dependent differences are observed in the sets of identified adhesins. In comparison to laboratory strains, hyperadhesive clinical isolates showed increased incorporation of cell wall adhesins, especially from Epa cluster I and Awp2 cluster V. Cluster V includes a group of 13 putative adhesins in reference strain ATCC 2001 (also named CBS138) (Xu et al., 2020) of which Awp2 and Awp4 seem widely present in cell walls of all analyzed strains whereas others (Awp8 - Awp11) have been encountered only in hyperadhesive isolates (Gómez-Molero et al., 2015). In recent work, we demonstrated the importance of Awp2 for hyperadhesiveness to plastic in two clinical isolates through functional characterization studies with deletion mutants (Reithofer et al., manuscript under review).

To further our knowledge on the role and importance of unknown cell wall adhesins in *C. glabrata*, here we focused on a clinical strain isolated from central venous catheter that forms high biofilm on polystyrene. As expected, proteomic analysis of its cell walls revealed abundant incorporation of adhesins and led to identification of novel adhesins. Phenotypic characterization of novel cluster III adhesin Awp14 did not confirm its importance for biofilm formation onto plastic but pointed to a subtle role in cell-cell interactions, possibly with chitin as binding ligand.

## Materials and Methods

### *C. glabrata* Strains

Hyperadhesive isolate PEU1221 was obtained from a central venous catheter (CVC) by routine clinical diagnostic procedure in University Medical Center Göttingen (Germany) and identified using MALDI-TOF (Bruker). Strain ATCC 2001 [karyotype I (Bader et al., 2012)] was used as reference strain. Unless mentioned otherwise, cultures grown overnight in liquid YPD (1% yeast extract, 2% peptone and 2% glucose) at 37°C were used as starting material for the assays described.

### Cell Wall Isolation and Mass Spectrometric Analysis

PEU1221 and ATCC 2001 were inoculated from overnight cultures in fresh YPD and grown at 37°C to logarithmic phase. From these cultures, 20 OD_600_ units in YPD were seeded in polystyrene Petri dishes (20 for PEU1221; 40 for ATCC 2001) in a volume of 20 mL per plate. After incubating for 24 h at 37°C in a moist environment, the settled cell layers were gently washed with milli-Q water to remove unbound cells, and adhered cells were collected by scraping. Cell wall isolations were performed using a Fastprep 24 instrument (MP Biomedicals) as described (De Groot et al., 2004; De Groot et al., 2008). Complete cell breakage was verified using a microscope. Cell walls were washed extensively with 1 M NaCl, and subsequently two times with SDS extraction buffer (50 mM Tris-HCl, 2% SDS, 100 mM Na-EDTA, 150 mM NaCl, and 0.8% β-mercaptoethanol, pH 7.8) at 100°C for 10 min. Finally, the cell walls were washed extensively with water, lyophilized, and stored at -20°C until use.

Preparation of cell walls and digestion with Trypsin Gold (Promega) for mass spectrometric analysis was performed as described (Yin et al., 2005). The peptide material was freeze-dried and taken up in 50% acetonitrile, 2% formic acid. After measuring the OD_214_ and calibrating against a peptide mix control sample (Thermo Scientific), samples were diluted in 0.1% trifluoroacetic acid solution to reach a peptide concentration of about 0.25-0.5 pmol µl^-1^. The amount of sample used per run was 0.5-1.0 pmol peptide material. Duplicate biological samples were analyzed using an amaZon Speed Iontrap with a CaptiveSpray ion source (Bruker) coupled to an EASY-nLC II (Thermo Scientific) chromatographic system, employing identical settings as described previously (Gómez-Molero et al., 2015). Precursor ions were automatically selected for low-energy collision-induced dissociation (CID) to obtain fragmentation spectra for peptide identification.

### MS/MS Database Searching

After processing the raw MS/MS data with Data Analysis software (Bruker), database searching was performed against *C. glabrata* protein sequences in NCBI using Mascot software (Version 2.5.1). Simultaneous searching was performed against a “common contaminants database” (compiled by Max Planck Institute of Biochemistry, Martinsried) to minimize false identifications. Mascot search parameters were: trypsin with allowance of one missed cleavage, fixed modification of cysteine with carbamidomethyl, variable modification of oxidized methionine, peptide charge states +2, +3, and +4, a peptide cut-off score of 20, peptide and MS/MS mass error tolerances of 0.3 Da, decoy database activated, and 1% false discovery rate as output threshold. Protein identifications based on a single peptide MS/MS match were only taken into consideration if the protein had been identified previously in cell walls of *C. glabrata*, and in such case, the validity of the identified peptide was verified by manual inspection of MS/MS spectra in the raw data using the Data Analysis software. Unmatched peptides were subjected to a second Mascot search with different settings: semitrypsin as the protease, including N/Q deamidation as variable modification, and a cut-off peptide score of 40. Identified semitryptic peptides solely served to extend the sequence coverage of proteins identified in the first trypsin search. Mass spectrometric details of identified individual peptides identified in both PEU1221 samples are given in **Supplementary Table S1**. The detailed proteomics data of the ATCC 2001 samples have already been presented previously (Gómez-Molero et al., 2015), therefore, ATCC 2001 data is here only used for comparing identified proteins in both strains (**Table 1**) and for peptide counting (**Table 2**).

**Table 1:** Identified cell wall proteins in biofilm cells of hyperadhesive *C. glabrata* isolate PEU1221, and comparison to reference strain ATCC 2001.

Proteins identified in PEU1221^a^			ATCC 2001^b^
Name	Acc. number (CGD/NCBI)	Structural information	
***Putative adhesins***			
Epa3	CAGL0E06688g/QHS65616	cluster I^c^; GPI	+
Epa6	CAGL0C00110g/QHS64812	cluster I; GPI	+
Epa7	CAGL0C05643g	cluster I; GPI	–
Epa22	CAGL0K00170g/QHS67889	cluster I; GPI	–
Awp14	CAGL0A04851g	cluster III; GPI	–
Awp2	CAGL0K00110g/QHS67887	cluster V; GPI	+
Awp2a/Awp8	CAGL0B00154g/QHS64595	cluster V; GPI	–
Awp2b/Awp9	CAGL0B05061g/QHS64811	cluster V; GPI	–
Awp2c/Awp10	CAGL0F00099g/QHS65618	cluster V; GPI	–
Awp2d/Awp11	CAGL0J12067g/QHS67886	cluster V; GPI	–
Awp2e	CAGL0H00209g/QHS66441	cluster V; GPI	–
Awp4	CAGL0M00121g/QHS69029	cluster V; GPI	+
Awp12	CAGL0G10219g/QHS66440	cluster VII; GPI	+
***Non-adhesin proteins - core proteome***			
*Carbohydrate-active enzymes*			
Crh1	CAGL0G09449g/QHS66407	GH16; GPI	+
Utr2	CAGL0C02211g/QHS64902	GH16; GPI	+
Gas1	CAGL0G00286g/QHS66009	GH72; GPI	+
Gas2	CAGL0M13849g/QHS69637	GH72; GPI	+
Gas4	CAGL0F03883g/QHS65772	GH72; GPI	+
Gas5	CAGL0F01287g/QHS65665	GH72; GPI	+
Scw4	CAGL0G00308g/QHS66011	GH17	+
*Putative non-enzymatic glucan crosslinkers*			
Cwp1.1^d^	CAGL0F07601g/QHS65929	Pir repeat; GPI	+
Cwp1.2	CAGL0F07579g/QHS65928	Pir repeat; GPI	+
Pir2	CAGL0I06182g/QHS67166	9 Pir repeats	+
Pir3	CAGL0M08492g/QHS69399	9 Pir repeats	+
Pir4	CAGL0I06160g/QHS67165	2 Pir repeats	+
Tir1	CAGL0F01463g/QHS65669	Srp1/Tip1 family; Pir repeat; GPI	+
*Phospholipases*			
Plb1	CAGL0J11770g/QHS67877	GPI	–
Plb2	CAGL0J11748g/QHS67876	GPI	+
*Unknown function*			
Ecm33	CAGL0M01826g/QHS69105	Ecm33 family; GPI	+
Pst1	CAGL0E04620g/QHS65527	Ecm33 family; GPI	+
Ssr1	CAGL0H06413g/QHS66715	GPI	+
Tir2	CAGL0F01485g/QHS65670	Srp1/Tip1 family; GPI	+

a See **Supplementary Table S1** for mass spectrometric details of a representative PEU1221 sample.

b Protein identification (+) or not (-) in reference strain ATCC 2001. Details of the ATCC 2001 analysis can be found in (Gómez-Molero et al., 2015).

c For adhesin cluster info see (De Groot et al., 2008; Xu et al., 2020).

d Presence in PEU1221 inferred from favorable Mascot assignment of unique but isobaric peptides present in Cwp1.1 and Cwp1.2.

**Table 2:** Semi-quantitative peptide-counting analysis of PEU1221 and ATCC 2001 cell wall proteomics data.

Peptides identified	PEU1221	ATCC 2001^b^
Total number of peptides	950	931
Core proteome (Cwp1)	76% (49%)	90% (56%)
Adhesins	24%	10%
**Number of adhesin peptides**	228	92
Cluster I	42%	16%
Cluster III (Awp14)	10%	–
Cluster IV^a^	0.4%	1%
Cluster V	43%	82%
Cluster VII	4%	1%

a Only a single non-unique cluster IV peptide was identified in both strains.

b Based on peptide data in Gómez-Molero et al. (2015).

### Generation of Deletion Mutants

*AWP14* deletion mutants were generated in hyperadhesive strain PEU1221 and reference strain ATCC 2001 using *SAT1*-flipper system plasmid pSFS1a (Reuß et al., 2004), designed in pBluescript II backbone for use in *C. albicans* but also functional in *C. glabrata*. This plasmid contains the *SAT1* flipper gene, conferring resistance to nourseothricin (NT), for transformant selection, and an inducible *FLP1* gene under control of the *CaSAP2* promoter for excision of the integrated cassette by recombination of palindromic flippase recognition sequences on both sides of the construct. Regions of about 0.6 kb upstream and within the *AWP14* coding sequence were amplified using proofreading KAPA polymerase and cloned into the *Kpn*I and *Xho*I (upstream fragment) and *Not*I and *Sac*I (downstream fragment) sites of the pSFS1a vector. Correctness of the deletion cassette was verified by Sanger sequencing. Genomic incorporation of this construct leads to deletion of 595 bp of the A-domain of *AWP14*. *C. glabrata* transformation was performed by electroporation of competent cells prepared using lithium acetate (De Backer et al., 1999), and transformants were selected on YPD agar containing 200 µg/mL NT. Deletion mutants among transformants were identified by PCR using combinations of primers that cover the integration junctions and combining external and internal *AWP14* primers. Deletion cassettes were removed from the genome by inducing the flippase gene by growth in YCB-BSA-YE (1.17% yeast carbon base, 0.4% bovine serum albumin, 0.2% yeast extract, pH 4.0) and selecting slow-growing colonies on plates containing a low concentration of 10 µg/mL NT. Loss of the deletion cassettes was verified by PCR (**Supplementary Figure S1**). In each background, at least two deletion mutants from independent transformation experiments were obtained and in all phenotypic assays data of mutants therefore represent the average of at least two mutants. Oligonucleotides used in this work are listed in **Supplementary Table S2**.

### Adhesion to Polystyrene

Adhesion to polystyrene was measured using two different assays depending on the time allowed to adhere, 4 or 24 h, the latter allowing more time for biofilm formation. For the 24 h experiment, overnight cultures in YPD at 37°C were adjusted to a cell density OD_600_ = 0.5 in fresh YPD, and 200 µL of the cell suspension was pipetted into a polystyrene 96-well plate and incubated at 37°C for 24 h in a humid environment. Unattached cells were removed by gentle washing with milli-Q water, and the remaining adhered cells were stained with 0.1% crystal violet (CV) solution for 30 min followed by washing with milli-Q water. Finally, CV was solubilized in 33% glacial acetic acid and quantified by measuring the OD_595_ using a microplate reader (Molecular Devices). Data for each strain are the average of six technical and at least two biological replicates.

For the 4 h adhesion experiment, overnight cultures were diluted to OD_600_ = 0.05 in phosphate-buffered saline (PBS), and 0.5 mL (~1 × 10^5^ cells) was pipetted in 12-wells plates and incubated for 4 h at 37°C. Unbound cells were removed by two washes with PBS, after which adhered cells were treated for 10 min at room temperature (RT) with a 2.5% porcine pancreas trypsin (Sigma) in PBS solution. Finally, PBS was added to a final volume of 0.5 mL, cells were resuspended and measured using a MACSQuant (Miltenyi Biotec) flow cytometer. Data are the average of at least two biological replicates measured in triplicate.

### Adhesion to Fungal Cell Wall and Extracellular Matrix Compounds

Binding capacity of *C. glabrata* cells to fungal cell wall components and extracellular matrix (ECM) collagen was also evaluated by flow cytometry following a similar procedure as described above for 4 h adhesion to polystyrene, using the same amount of cells. However, before incubation with *C. glabrata*, the microtiter plates were coated with chitin (Sigma; 250 μg/mL in 1% acetic acid), laminarin (β-1,3-glucan, Thermo Fisher; 500 μg/mL in milli-Q water), pustulan (β-1,6-glucan, Calbiochem; 500 μg/mL in 50 mM potassium acetate buffer) or bovine collagen (Sigma; 250 μg/mL in 0.2 M bicarbonate buffer, pH 9.6) by adding 500 μL of the respective solutions, and allowing for passive adsorption (1 h at 30°C followed by overnight incubation at 4°C), in the case of chitin and collagen, or evaporation (overnight at 37°C), in the case of laminarin and pustulan.

### Growth Rate and Sensitivity to Zymolyase

For determination of growth rates, cells from overnight cultures were inoculated 1/100 in fresh YPD and incubated in 200 µL volumes in 96-well plates at 37°C with agitation in a SpectraMax 340PC microplate reader. The increase in OD_600_ was followed in time. For determination of zymolyase sensitivity, cells were grown in YPD until log phase, collected, and resuspended at an OD_600_ of 2.0 in 10 mM Tris-HCl, 0.25% of β-mercaptoethanol, pH 7.5. After 1 h of incubation at RT, 180 µL of cell suspensions and 20 µL of up to 100 U/mL zymolyase 20T were mixed in a 96-well plate and placed in the microplate reader at 37°C. Decrease in OD_600_ was measured each minute after a short mixing pulse. Curves represent averages of five technical and two biological replicates.

### Sedimentation and Aggregation

Overnight cultures in YPD at 37°C were transferred to glass tubes. Every 10 min 20 µL of sample was carefully taken 2 cm below the surface of the cell suspension for OD_600_ measurements. Cell aggregation of overnight cultures in YPD was observed with a Leica DM1000 microscope mounted with a MC170 HD digital camera and by flow cytometry measuring size (FSC-A) and granularity (SCC-A) of 20,000 cell particles.

### Cell Surface Hydrophobicity

Overnight cultures in YPD at 37°C were washed two times with PBS and resuspended at an OD_600_ of 0.7. Cell suspensions were mixed with hexadecane (Sigma) in glass tubes at a 15:1 volume ratio. Upon 1 min of gentle vortexing, the phases were allowed to settle for 10 min after which the OD_600_ of the aqueous phase was measured. Each strain was assayed twice with two technical replicates each.

### Drug Sensitivity Assays

Susceptibility to antifungal agents was tested in 96-well plates following EUCAST guidelines (EUCAST, 2020). Twofold serial dilutions of compounds were prepared in YPD and mixed 1:1 with cells from overnight cultures diluted to an OD_600_ of 0.01. Plates were incubated for 24 h at 37°C. Minimal inhibitory concentrations (MIC) were determined after reading the OD_600_ in a microplate reader. Antifungals were Caspofungin (Merck Sharp & Dohme), Micafungin (Astellas), and Nikkomycin Z (Sigma). Sensitivity to cell wall perturbants Calcofluor white (CFW) and Congo red (CR) was tested in spot assays. Tenfold serial dilutions were prepared from overnight cultures, and 4 µL were spotted on YPD plates containing 100 µg/mL CFW or 100 µg/mL CR. Growth was monitored after 24 and 48 h of growth at 37°C.

### Statistical Analysis

Statistical significance of phenotypic data was analyzed by Student’s t-tests or one-way ANOVA followed by *post hoc* Delayed Matching to Sample (DMS) tests. P values <0.05 were considered statistically significant.

### Bioinformatics and Protein Structural Modeling

N-terminal ends of identified CWPs produced by signal peptide cleavage were analyzed using SignalP (http://www.cbs.dtu.dk/services/SignalP/). The three-dimensional structure of the ligand-binding or A-domain of Awp14 (residues 22-400) was modeled using ColabFold (Mirdita et al., 2021) and visualized using iCn3D (Wang et al., 2020). Structural similarity to proteins in the PDB database was analyzed using the PDBeFold, with a default cut-off for lowest acceptable similarity match of 70% (Krissinel and Henrick, 2004), and DALI (Holm, 2020) servers.

## Results

### PEU1221, a Clinical Isolate From Central Venous Catheter With High Biofilm-Forming Capacity

**Figure 1:**
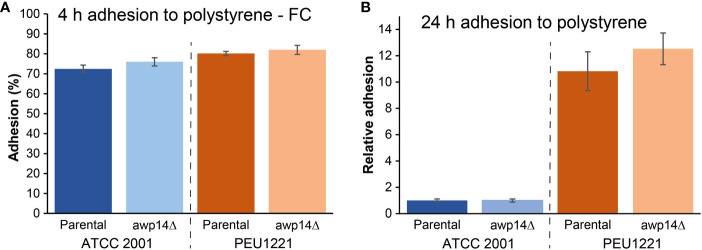
Awp14 is not responsible for the HBF phenotype of PEU1221. **(A)** Adhesion to polystyrene after 4 h measured by flow cytometry (FC). **(B)** Binding to polystyrene after 24 h measured by Crystal violet (CV) staining. *AWP14* was deleted and analyzed in two genetic backgrounds: reference strain ATCC 2001 and HBF strain PEU1221.

*C. glabrata* strain PEU1221 was isolated from an infected central venous catheter during routine diagnostic procedure. When measuring adhesion to polystyrene after 4 h of incubation in PBS using flow cytometry, only slightly higher adhesion was observed for PEU1221 compared to reference strain ATCC 2001, however, PEU1221 showed an about tenfold higher amount of adhered biomass onto polystyrene after 24 h of incubation in YPD measured by Crystal violet staining, demonstrating the high biofilm-forming (HBF) capacity of this clinical isolate (**Figure 1**).

### The Cell Wall Proteome of PEU1221: A Core Proteome and Increased Incorporation of Adhesins

Previous studies have revealed correlations between the capacity to form biofilms and the incorporation of cell wall adhesins in *Candida* (Gómez-Molero et al., 2015; Moreno-Martínez et al., 2021). This prompted us to investigate the incorporation of cell wall adhesins in strain PEU1221 in comparison to ATCC 2001, a low-biofilm-forming (LBF) strain, using a proteomic approach. Cell walls of PEU1221 and ATCC 2001 were purified from 24 h biofilms on polystyrene, subjected to trypsin digestion, and analyzed by tandem mass spectrometry, presented in **Tables 1**, **2**. Consistent with previous analyses of other strains (Gómez-Molero et al., 2015), from comparing the cell wall proteomes of the two strains it is clear that the identified proteins can be divided in two groups: (i) a stable core wall proteome present in both strains, and (ii) a strain-dependent variable set of putative adhesins that is amplified in the HBF strain. The identified non-adhesin core wall proteome consisted of 19 proteins (**Table 1**), including carbohydrate-active enzymes from Crh, Gas, and Bgl2/Scw4 families involved in cell wall polysaccharide remodeling, non-enzymatic proteins of Cwp1, Pir, and Srp1/Tip1 families with suggested roles in crosslinking of β-glucan chains, Plb proteins with presumed lysophospholipase activity, and proteins with unknown functions including widely present Ecm33 family proteins and Ssr1. Except Plb1, which was identified only in PEU1221, all core wall proteins were identified in both strains (**Table 1**). Counts of identified MS/MS spectra showed that the Cwp1 family proteins Cwp1.1 and Cwp1.2 account for about half of all the identified peptides and thus are by far the most abundant proteins in cell walls of both strains (**Table 2**). For more information on these core wall proteins, we refer to our previous studies (De Groot et al., 2008; Gómez-Molero et al., 2015).

In contrast to the core wall proteome, the proteomic data showed a clearly elevated incorporation of adhesins in strain PEU1221. Thirteen different adhesins were unambiguously identified in PEU1221 compared to only five in ATCC 2001 biofilms (**Table 1**). Consistent with this, the number of different peptides identified from adhesins in PEU1221 (78 peptides, see **Supplementary Table S1**) was also higher than in ATCC 2001 [23 peptides, for details, see Gómez-Molero et al. (2015)]. Moreover, adhesin peptides represented 24% of the total amount of identified peptides in PEU1221, higher than the 10% in ATCC 2001 (**Table 2**). Altogether, our proteomic data indicate that incorporation of adhesin protein molecules was about two- to three-fold higher in cell walls of HBF strain PEU1221 than in LBF strain ATCC 2001 under the tested biofilm-forming conditions.

### Adhesins in the Cell Wall of Strain PEU1221

Breaking down the adhesins in different families, Epa cluster I adhesins identified in PEU1221 were Epa3, 6, 7 and 22, whereas in ATCC 2001 only Epa3 and 6 were unambiguously identified. Epa22 was unambiguously identified for the first time in *C. glabrata* while Epa7 has been identified before in two hyperadhesive isolates (Gómez-Molero et al., 2015). Both the total number of Epa peptides and their proportion among all adhesin peptides (42% of 228 in PEU1221 versus 16% of 92 in ATCC 2001, **Table 2**) was higher in PEU1221 than in ATCC 2001. This is in accordance with the documented upregulation and importance of *EPA* genes, in particular *EPA6* and *EPA7*, during biofilm formation (Iraqui et al., 2005).

**Figure 2:**
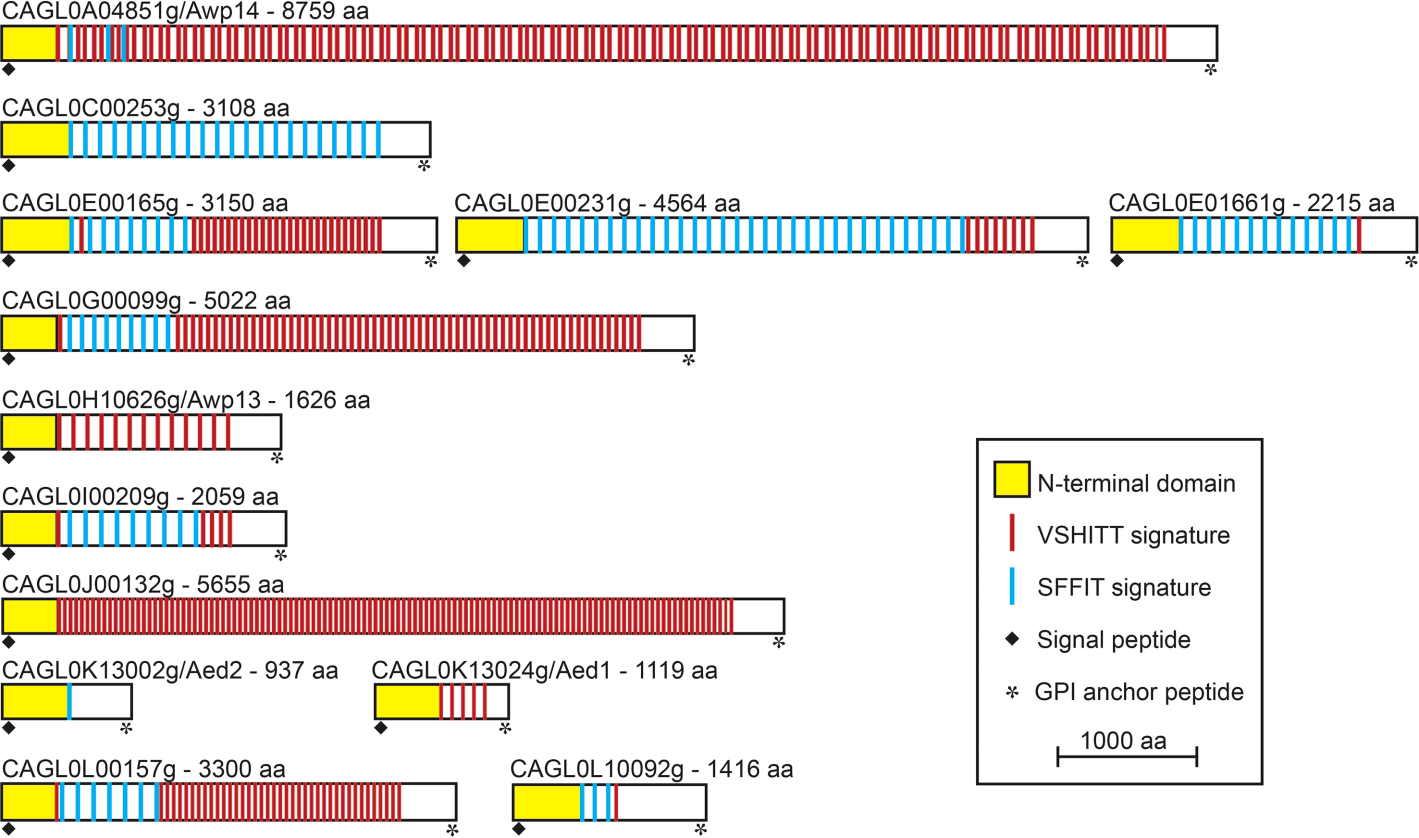
Modular organization of the 13 GPI-modified cluster III cell wall adhesins in *C. glabrata* ATCC 2001. In all proteins, an N-terminal ligand-binding domain is followed by a C-terminal low complexity region containing megasatellites (large repeats) with VSHITT or SFFIT cores. Proteins are organized by their chromosomal localization and their sizes are to scale.

PEU1221 cell walls also showed increased incorporation of cluster V adhesins: whereas Awp2 and 4 were identified in both strains, Awp8, 9, 10, 11, and Awp2e were identified only in PEU1221. Awp8 - 11 have also been identified previously in other HBF clinical isolates (Gómez-Molero et al., 2015), but Awp2e, was identified for the first time. Adhesin cluster VII protein Awp12 was identified in both strains albeit with four different peptides in PEU1221 and only with a single peptide in ATCC 2001. Finally, another putative adhesin that was only detected in PEU1221 is CAGL0A04851g. This cluster III protein, which we named Awp14, was identified for the first time in cell walls of *C. glabrata*. Cluster III includes 13 putative adhesins (**Figure 2**), mostly uncharacterized, of which only Aed1 and Awp13 have been previously identified in cell walls by mass spectrometry. The total (23) and different (10) number of Awp14 peptides identified suggests it is one of the most abundant adhesins in PEU1221 biofilm cells. We therefore focused our further studies on functional characterization of this protein.

### Characterization of Cluster III Adhesin Awp14

*AWP14* is located on chromosome A, with its 3` end close to the right-hand telomere, and spans more than 26 kb according to the ATCC 2001 genome update published by Xu and colleagues (Xu et al., 2020). However, this published sequence appears to contain a sequencing error (in a repeat region), leading to premature translation termination. By comparing to other repeat sequences and to the more recently published BG2 genome (Xu et al., 2021), the error is corrected by inserting a T at position 15,180, resulting in an extremely large protein of 8,759 aa. Like all putative *C. glabrata* adhesins, this protein sequence starts with a signal peptide for secretion and ends with a GPI anchor addition signal, both removed during its translocation to the cell surface as result of processing steps. The remaining mature protein consists of an A-domain of about 375 aa, immediately followed by an extremely long tail comprising megasatellites (repeat sequences) that contain VSHITT (114) or SFFIT (4) cores, which are present in all cluster III adhesins (**Figure 2**) as well as in adhesins from other clusters (De Groot et al., 2013).

**Figure 3:**
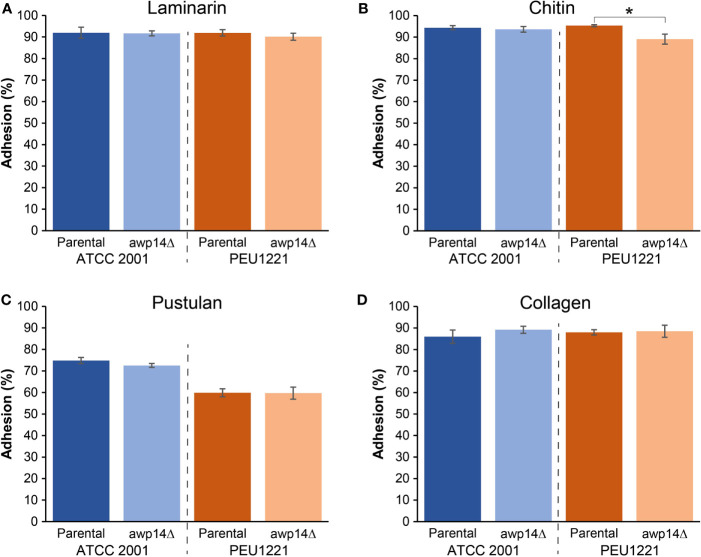
Deletion of *AWP14* results in a slight reduction of HBF strain PEU1221 binding to chitin. Binding to fungal cell wall (β-1,3-glucan, β-1,6-glucan, and chitin) and mammalian extracellular matrix (collagen) molecules was measured by flow cytometry after incubating *C*. *glabrata* cells for 4 h in microtiter plates containing prefixed layers of **(A)** laminarin (β-1,3-glucan), **(B)** chitin, **(C)** pustulan (β-1,6-glucan), and **(D)** collagen. The statistically significant difference in chitin binding between PEU1221 and mutant is indicated by an asterisk.

We generated *AWP14* deletion mutants in both PEU1221 and reference strain ATCC 2001. To avoid problems with correct integration of the deletion cassette and PCR verification of the mutants, we deleted the largest part of the A-domain but staying away from repeat sequences in the C-terminal domain. Obtained deletion mutants were phenotypically characterized, thereby mostly focusing on cell surface-related features. First, we determined if deletion of *AWP14* led to altered adhesion or biofilm formation on polystyrene. However, neither in ATCC 2001 nor PEU1221 background significant changes were observed upon deleting *AWP14* (**Figure 1**), indicating that Awp14 is not responsible for the high biofilm formation of the latter. Then, we tested if the protein might perhaps play a role in cell-cell interactions with neighboring cells through binding to cell wall molecules by measuring cells extracted after binding to laminarin (β-1,3-glucan), chitin, and pustulan (β-1,6-glucan). In these flow cytometry assays, the *awp14*Δ mutants did not show alterations in binding to glucan, however, a small but significant decrease (95% versus 89%) in binding to chitin was observed for the *awp14*Δ mutants in PEU1221 background (**Figure 3**). In a similar assay, we also tested binding to collagen as model for possible host interactions but found no effect of deleting *AWP14*. Further, except for pustulan, these *in vitro* assays did not show differences in affinity between ATCC 2001 and PEU1221, indicating that the different biofilm-formation capacities of the two strains do not affect their binding to these cell surface molecules. For pustulan, however, a lower binding for PEU1221 (60%) than for ATCC 2001 (75%) was observed, and this was not affected by deleting *AWP14*.

**Figure 4:**
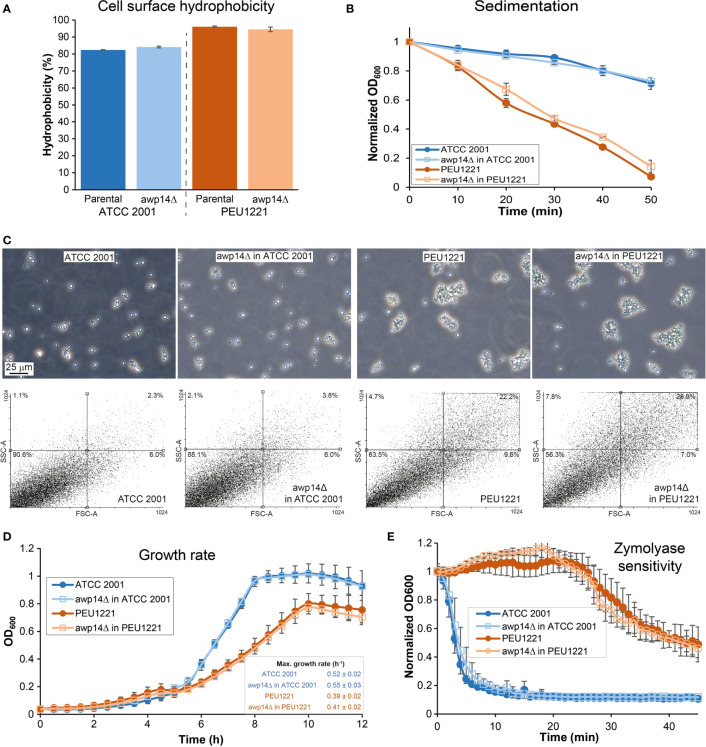
Elimination of *AWP14* does not cause (major) alterations in cell surface-related properties in PEU1221 and ATCC 2001. **(A)** Cell surface hydrophobicity. **(B)** sedimentation, **(C)** aggregation, **(D)** growth rate, and **(E)** zymolyase sensitivity of parental strains and *awp14Δ* mutants in PEU1221 and ATCC 2001. **(C)** Aggregation was analyzed by microscopy (Top panel) and flow cytometry (Bottom panel) by measuring 20,000 cell particles per strain. FSC-A, particle size; SSC-A, particle complexity. Percentage of cell particles in each quadrant are indicated. Note the increased dot population in the right upper part of the dotblots in PEU1221 background strains indicating cell aggregation.

Previous work has shown that HBF phenotypes may coincide with increased cell surface hydrophobicity, increased cell-cell aggregation, and faster sedimentation in liquid (Gómez-Molero et al., 2015; Moreno-Martínez et al., 2021). This is also the case for PEU1221. It has higher surface hydrophobicity, sediments faster, and showed more cell clumping than the reference strain as observed by microscopy and flow cytometry (**Figure 4**). Probably because of the cell clumping, OD_600_ measurements of liquid cultures indicate lower growth rates during exponential phase and reached a lower maximum cell density. Deleting *AWP14* did not have an evident effect on these phenotypes albeit that in PEU1221 background the *awp14*Δ mutant appeared to sediment slightly slower than the parental strain and flow cytometry indicated increased complexity but not size of the measured cell particles. Further cell wall-related phenotypes studied were minimal inhibitory concentrations of the antifungal compounds caspofungin, micafungin, and nikkomycin using microbroth dilutions, growth inhibition by Calcofluor white and Congo Red in spot assays, and resistance to cell lysis by zymolyase (a β-1,3-glucanase-containing enzyme preparation). These assays did not detect any phenotype caused by deleting *AWP14* (not shown) but noteworthy is the high resistance to zymolyase of the HBF strain as compared to ATCC 2001 (**Figure 4E**). Similar higher resistance to zymolyase as compared to ATCC 2001 has also been observed previously for other HBF strains (Gómez-Molero et al., 2015), however, what architectural cell wall changes underlie this phenotype and if this is related to adhesin incorporation, is currently unknown.

### Structural Modeling of the Awp14 A-Domain Reveals a β-Helix Structure

**Figure 5:**
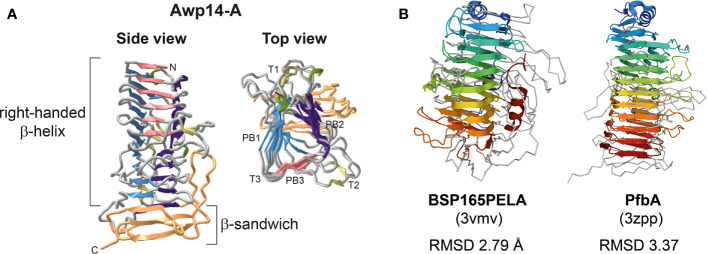
The modeled 3D structure of the N-terminal ligand-binding domain of Awp14 shows a β-helix structure with similarity to bacterial polysaccharide lyases and adhesins. **(A)** Cartoon presentations of the 3D structural model of the Awp14 A-domain (residues 22 - 400). Indicated are the β-helix and C-terminal β-sandwich structure. The three β-strands forming a single turn in β-helices are referred to as PB1, PB2, and PB3, and the loops between them are labeled T1, T2, and T3 (Yoder and Jurnak, 1995). **(B)** Structural superposition of the Awp14 A-domain (grey ribbon) with bacterial β-helix proteins colored from the N-terminus (blue) to the C-terminus (red): the secreted pectate lyase Bsp165PelA from *Bacillus* sp. N16-5 (Zheng et al., 2012), and the wall-associated adhesin PfbA from *S. pneumoniae* (Suits and Boraston, 2013). The RMSD values after superposition were calculated for the respective β-helix domains by PDBeFold.

Finally, the 3D structure of the Awp14 A-domain was predicted using ColabFold (**Figure 5**). The obtained structural model revealed a right-handed β-helix containing three parallel β-sheets per coil followed by a C-terminal β-sandwich structure. Similarity searches against the PDB database revealed several proteins with similar three-faced β-helices, including a variety of polysaccharide lyases from different bacteria [e. g. the pectate lyase Bsp165PelA from *Bacillus* sp. N165 (Zheng et al., 2012) and alginate lyase FPU-7 from *Paenibacillus* sp. str. (Itoh et al., 2019)], adhesin HMW1 (Yeo et al., 2007) and heme-hemopoxin binding HxuA (Baelen et al., 2013) from *Haemophilus influenza*, and the cell-wall attached adhesin PfbA (plasmin- and fibronectin-binding protein A) of *Streptococcus pneumoniae* (Suits and Boraston, 2013). Root mean square deviation (RMSD) values between Awp14-A and the search results ranged from 2.79 to 5.92 Å, indicating high structural similarity.

## Discussion

*C. glabrata* contains a large number of putative cell wall adhesins that are thought to mediate fungus-host interactions and biofilm formation onto abiotic medical surfaces and thus are considered important virulence factors. However, of the about 70 adhesins discovered by genomic studies, only the Epa cluster of about 20 proteins has been well-characterized. Epa1 was first shown to play a crucial role in binding to human epithelial cells (Cormack et al., 1999). In later studies, members of the family were shown to have lectin activity and to bind to terminal galactoside residues on host cell surface proteins (Zupancic et al., 2008; Maestre-Reyna et al., 2012; Diderrich et al., 2015; Hoffmann et al., 2020). To date, the role of the other putative adhesins remains poorly understood. Phylogenetic analysis indicated that the seven adhesins of cluster II share similarity with Epa proteins in their ligand-binding - so-called PA14 - domains (De Groot et al., 2008). However, the supposed lectin activity yet has to be demonstrated, and cluster II adhesins have never been encountered in cell wall preparations.

Previous proteomic studies with clinical isolates in *C. glabrata* and *C. parapsilosis* showed that their cell wall proteomes consist of a stable core proteome and a varying number of incorporated cell wall adhesins. More than by changing growth conditions, notable plasticity in incorporation of wall adhesins was observed when comparing strains with differences in their capacity to form biofilm. In both species, a positive correlation was observed between the biofilm-forming capacity of isolates and the incorporation of adhesins (Gómez-Molero et al., 2015; Moreno-Martínez et al., 2021). PEU1221 is a clinical *C. glabrata* strain isolated from an infected venous catheter and was shown to have an about tenfold higher capacity to form biofilm onto polystyrene than reference strain ATCC 2001. Intending to shed more light on the functionality of unstudied adhesins, we targeted cell walls of strain PEU1221 using a proteomic approach.

Compared to reference strain ATCC 2001, proteomic analysis of biofilm cells showed increased incorporation of adhesins in PEU1221, confirming the earlier noted correlation between biofilm-forming capacity and adhesin incorporation in *Candida* (Gómez-Molero et al., 2015; Moreno-Martínez et al., 2021). Not only the relative abundance of adhesin peptides was higher in strain PEU1221, also more different adhesin peptides and proteins were identified. Of the eight adhesins that were identified only in PEU1221, five previously have also been identified (only) in HBF clinical isolates (Gómez-Molero et al., 2015). Interestingly, three of the proteins (Epa22, Awp14 and Awp2e) that were identified only in PEU1221, were identified for the first time in cell walls of *C. glabrata*. One may argue that our proteomic analysis of an unsequenced strain such as PEU1221 might be incomplete as it depends on protein sequences present in the public databases while genomic studies have shown strain-dependent variations in adhesin genes (Vale-Silva et al., 2017; Carreté et al., 2018; Xu et al., 2021). However, observed genomic variations are mostly limited to the C-terminal glycosylated repeat regions of the adhesins whereas our proteomic analysis only identifies unglycosylated peptides from the N-terminal ligand-binding domains. Further, the almost equal number of cell wall peptides (931 vs. 950) identified in both strains also indicates that this is not an issue.

Because of its relative abundance (in PEU1221), subsequent functional characterization work was concentrated on cluster III adhesin Awp14. Adhesin cluster III comprises 13 GPI-CWPs (De Groot et al., 2013; Xu et al., 2020) (**Figure 2**). These proteins share the typical modular adhesin protein structure with N-terminal domains of 300 - 400 amino acids that probably define the ligand-binding properties, followed by large low-complexity spacer domains that present the N-terminal domains at the cell surface. The spacer regions of cluster III proteins in ATCC 2001 are very large (~3,000 residues on average) and in Awp14 exceeds 8,000 amino acids. Further, these regions are rich in serine (S) and threonine (T) residues, and thus contain many potential acceptor sites for *O*-glycosylation, and they are spiked with large internal repeats that contain VSHITT or SFFIT cores (Thierry et al., 2010; De Groot et al., 2013). Only Awp13 and Aed1 of this adhesin cluster have been identified previously in purified walls by proteomic studies; Awp13 in biofilm cells of HBF strain PEU427 (Gómez-Molero et al., 2015), and Aed1 in planktonic stationary phase cells of ATCC 2001 that were grown in medium with low (0.36%) glucose (Kraneveld et al., 2011). Deletion of *AED1* has been reported to result in a reduction of adherence to human umbilical vein endothelial cells, but the molecular mechanism remained unresolved (Desai et al., 2011).

Adhesion and biofilm formation tests on polystyrene with *awp14*Δ mutants in both genetic backgrounds showed that the protein is not responsible for the HBF phenotype of PEU1221. Perhaps this is not so surprising as the level of cluster I (Epa family) and cluster V (Awp2 family) adhesins is also increased in the HBF strain. Furthermore, deletion of *AWP14* might also lead to compensatory upregulation of other adhesins, including related cluster III adhesins, possibly by affecting their subtelomeric silencing. As explained in the Results section, the *AWP14* deletion mutants lack the crucial gene part encoding the entire effector (or ligand-binding) domain. Thus, although we cannot fully exclude any functionality of the remaining part, it is unlikely that lack of drastic (adhesion) phenotypes would be caused by the fact that not the whole gene is deleted.

Apart of the HBF phenotype, isolate PEU1221 also shows other cell surface-related phenotypes that are typical of HBF strains, such as increased surface hydrophobicity, aggregation, rapid sedimentation, slower growth, decreased zymolyase sensitivity, and decreased binding to β-1,6-glucan in comparison to the reference strain. Most of these phenotypes were not affected by disrupting *AWP14*. However, sedimentation seemed slightly slower and cell aggregates showed slightly increased complexity (but not size) in the *awp14*Δ mutants in PEU1221. In addition, *in vitro* binding to chitin was slightly decreased by deleting *AWP14* in PEU1221. Altogether, these data suggest that Awp14 and other cluster III adhesins may have some role in self or non-self cell-cell interactions, possibly through binding to chitin molecules. In this respect, *in vitro* binding of *C. glabrata* to *C. albicans* hyphae mediated by cell wall adhesins, with possible implications in development of oropharyngeal candidiasis, has been reported (Tati et al., 2016).

In a recent study, we have resolved crystal structures of the ligand-binding domains of cluster VI proteins Awp1 and Awp3b, and performed homology modeling with cluster V protein Awp2 (Reithofer et al., manuscript under review). Remarkably, these domains were shown to consist of a right-handed β-helix followed by a β-sandwich structure, very similar to the obtained 3D model of the Awp14 A-domain. As found for Awp1, Awp2, and Awp3b, searches with the Awp14-A model against PDB yielded proteins with similar three-faced right-handed β-helix structures, including polysaccharide lyases (Zheng et al., 2012; Itoh et al., 2019) and adhesins from different bacteria (Yeo et al., 2007; Suits and Boraston, 2013), supporting their structural similarity, Thus, despite the low level of sequence identity of only 15% to 18% between Awp14-A and the ligand-binding domains of these cluster V and VI proteins, here we establish for the first time a structural relationship between the ligand-binding domains of adhesins from cluster III and those from clusters V and VI. Characterization of deletion mutants in our previous study showed that Awp2 is involved in biofilm formation on polystyrene. This was not the case for Awp1 and Awp3b (Reithofer et al., manuscript under review), which is consistent with the fact that they have never been identified in walls of HBF isolates (Gómez-Molero et al., 2015). However, further detailed functional characterization studies of these adhesin families are needed to elucidate their real contribution to biofilm formation and virulence and to identify their ligands.

## Data Availability Statement

The raw data supporting the conclusions of this article will be made available by the authors, without undue reservation, to any qualified researcher. Raw proteomics data will be made available in the PRIDE database.

## Author Contributions

Conceptualization, EE, OB, and PG. Funding acquisition, EE and PG. Investigation, JF-P, MA, EG-M, HD, and MB-M. Resources, HD, EE, OB, and PG. Supervision, OB and PG. Writing–original draft, JF-P, MA, and PG. Writing–review and editing, EE, OB, and PG. All authors have read and agreed to the submitted version of the manuscript.

## Funding

This work was supported by grants [SBPLY/19/180501/000114 and SBPLY/19/180501/000356] funded by the Regional government of Castilla-La Mancha and grants [SAF2013-47570-P, SAF2017-86188-P, and PID2020-117983RB-I00] funded by MCIN/AEI/10.13039/501100011033 and by ERDF A way of making Europe.

## Conflict of Interest

The authors declare that the research was conducted in the absence of any commercial or financial relationships that could be construed as a potential conflict of interest.

## Publisher’s Note

All claims expressed in this article are solely those of the authors and do not necessarily represent those of their affiliated organizations, or those of the publisher, the editors and the reviewers. Any product that may be evaluated in this article, or claim that may be made by its manufacturer, is not guaranteed or endorsed by the publisher.
