# Supplementary material for: Characterization of Awp14, A Novel Cluster III Adhesin Identified in a High Biofilm-Forming Candida glabrata Isolate
Source: Front Cell Infect Microbiol. 2021 Nov 15;11:790465. doi: 10.3389/fcimb.2021.790465 (PMC8634165; doi:10.3389/fcimb.2021.790465)
Supplement: Supplementary file 1 [file DataSheet_1.pdf]

SUPPLEMENTARY TABLE S1. MS/MS identification of cell wall proteins in hyperadhesive *C. glabrata* isolate PEU1221.

Protein(s) containing the identified peptide / NCBI/CGD accesion number	aa before	Identified peptide sequence[b]	aa after	Functional class	Peptide mass (m/z)	Mascot searches: Trypsin Score[a]	Trypsin #[a]	Semitrypsin & deamidation Score	Semitrypsin & deamidation #
Epa3 / CAGL0E06688g	F	TMILYGYFK	A	Adhesin cluster I Epa	1135			47	1
	K	VSLFLEK	D		834	40	1		
Epa6 / CAGL0C00110g	R	DNDGALSLTLK	T	Adhesin cluster I Epa	1146	91	6		
	L	FDTLPQAYNYR	T		1387			49	1
	M	GCTSLFDTLPQAYNYR	T		1905			80	1
	K	NGLSMELYSYDYLK	S		1695	95	1		
	K	NGLSMELYSYDYLK Deamidated (NQ)	S		1696			100	7
	T	SLFDTLPQAYNYR	T		1587			49	1
	C	TSLFDTLPQAYNYR	T		1688			61	2
	K	VENVAGNINFYYHAPMGC	T		2055			106	3
Epa7 / CAGL0C05643g	R	EYDAELSLTLK	T	Adhesin cluster I Epa	1281	66	8		
	K	NGLSMELYSYDLR	K		1560	58	2		
	K	NGLSMELYSYDLR Deamidated (NQ)	K		1561			53	1
Epa22 / CAGL0K00170g	K	DVYYPIR	M	Adhesin cluster I Epa	925	34	2		
	K	VSLNLEK	D		801	49	5		
Epa3 and Epa22	K	LGEYVAYDIWHVDSSK	N	Adhesin cluster I Epa non-unique peptides	1881	98	7		
Epa3 and Epa22	R	MFFNNIGK	D		970	54	3		
Epa3 and Epa22	R	MFFNNIGK Oxidation (M)	D		986	28	1		
Epa3, 6, 7, Epa22 and Epa14a/CAGL0L13552g	N	FGAGNAFDCCK	R		1246			74	2
Epa3, 6, 7, 14a, 22 and Epa19/CAGL0A00099g	F or L	GAGNAFDCCK	R or A		1099			40	1
Epa6 and 7	Y	AVWGSQTAK	T		946			50	4
Epa6 and 7	K	DDLTVHLDAGLYYPIR	I		1860	90	25		
Epa6 and 7	R	ESSADDFGNYQAY	A		1466			77	2
Epa6 and 7	R	ESSADDFGNYQAYAVWGSQTAK	D		2394	46	1		
Epa6 and 7	R	ESSADDFGNYQAYAVWGSQTAK Deamidated (NQ)	D		2395			52	1
Epa6, 7, Epa8/CAGL0C00847g, Epa11/CAGL0L13299g, Epa12/CAGL0M00132g and Epa13/CAGL0L13332g	R	IFFNNR	D		809	28	3		
Epa7 and Epa1/CAGL0E06644g	K	VDGVTGNINFYYHATK	G		1798	68	4		
Awp14 / CAGL0A04851g	K	APYNSPLVILGTLEVLR	S	Adhesin cluster III	1854	72	1		
	T	FEDDELLVLK	N		1220			65	1
	K	FSGIHIGGTYK	A		1179	29	3		
	K	GTDNVFIR	T		921	62	2		
	R	IDLGPDKIDENGVFTMK	N		1891	51	2		
	K	LLNLQAR	L		827	53	3		
	K	RPQYSMVINGK	V		1292	28	3		
	K	SFSIIGDR	K		893	55	1		
	R	TDDGYFDDNLFR	I		1477	85	4		
	K	YLDWGFSHATTK	F		1425	77	3		
Awp6 / CAGL0G10175g and Awp7 / CAGL0C00209g	R	VNMIELQAYLADIK	G	Adhesin cluster IV	1620	82	1		
Awp2 / CAGL0K00110g	K	GGALYYINNDK	Q	Adhesin cluster V	1227	43	1		
	K	GTSFEKPQR	L		1049	49	7		
	K	IHGISYDNYNFVR	Y		1597	68	2		
	R	SISPGSWTIK	D		1075	24	1		
	F	VFLNEGTVVVDDR Deamidated (NQ)	R		1463			65	8
Awp2a/Awp8 / CAGL0B00154g	K	GGALYYVNNDER	G	Adhesin cluster V	1370	32	1		
Awp2b/Awp9 / CAGL0B05061g	K	GGSLYYLNNGLQGFDIESK	H	Adhesin cluster V	2074	33	1		
	K	GGSLYYLNNGLQGFDIESK Deamidated (NQ)	H		2075			60	2
	K	GTSANMPQILK	L		1159	56	1		
	K	HALFVSFVFR	N		1222	72	1		
Awp2c/Awp10 / CAGL0F00099g	K	GTDANTPQILK	I	Adhesin cluster V	1157	55	5		
	K	NPIEGVGCVTVGENSVFNIK Deamidated (NQ)	D		2133			83	1
	K	STSVGVWK	I		863	37	3		
	K	SVTNTGFIYSK	G		1216	69	3		
	R	YNGSPPNSAPSICR	P		1519			67	5
	R	YNGSPPNSAPSICR Deamidated (NQ)	P		1520			46	2
Awp2d/Awp11 / CAGL0J12067g	K	GTSSSKPQQLK	I	Adhesin cluster V	1160	43	8		
Awp2e / CAGL0H00209g	K	GGALYYINDYLSGF	S	Adhesin cluster V	1552			92	1
	R	KSTSPGSWTIK	D		1191	35	1		
	Y	LAFQDMNTVPLYQTVR	V		1895			59	1
	F	QDMNTVPLYQTVR	V		1564			60	1
	K	STSPGSWTIK	D		1063	39	4		
Awp4 / CAGL0M00121g	K	ACVEIPLYTFK	V	Adhesin cluster V	1340	53	2		
	K	GKPPQSAPSICK	A		1269	58	15		
	K	SFTYDSLTGIIK	F		1344	91	3		
Awp4 and CAGL0D00143g	K	GTNAARPQQLK	I		1183	40	4		
Awp4 and CAGL0D00143g	K	INDGSFTNTGNIMFTSSQ Deamidated (NQ)	G		1934			113	1
Awp4 and CAGL0D00143g	R	LSTSAGSWK	I		935	56	3		
Awp4 and CAGL0D00143g	R	VNQGGALYYINNNLK Deamidated (NQ)	G		1681			68	1
Awp2a and Awp2d (ST: Awp2)	R	NEGTVVVDDR	Q		1103	40	1		

Awp2b (ST: Awp2e and CAGL0L00227g)	R	NDGTVVVDDR	N	Adhesin cluster V non-unique peptides	1089	72	1		
Awp2b and Awp2d	N	SVPMYQTVR	V		1080			41	1
Awp2c (ST: AWP4 and CAGL0D00143g)	K	GGALYYINNNLK	G		1339	52	2		
Awp2e or CAGL0L00227g	F	VFQNDGTVVVDDR	K		1463			99	7
Awp2e or CAGL0L00227g	F	VFQNDGTVVVDDR Deamidated (NQ)	K		1464			59	1
Awp2e or CAGL0L00227g	F	VFQNDGTVVVDDRK	S		1591			53	3
Awp12 / CAGL0G10219g	K	EITYNGNVQLDSYFLLK	A	Adhesin cluster VII	2016	50	1		
	K	EITYNGNVQLDSYFLLK Deamidated (NQ)	A		2017			96	1
	R	SGILVVHTTK	N		1054	62	7		
	N	WLHTGLFPGR	S		1183			46	1
Crh1 / CAGL0G09449g	K	ALATSFSENFTSESK	W	CaZy GH16	1618	96	1		
	K	AVDGEINGR Deamidated (NQ)	Y		931			47	2
	K	EYTYGDQSGSWQSIK	A		1748	72	5		
	R	FDNPSLK	S		819	43	1		
	R	GEFHGVNQPQK	E		1240	55	5		
	K	GNTATYDR	G		897	50	7		
	K	IEYTSDGLAMSLTK	R		1528	117	6		
	K	RFDNPSLK	S		976	53	6		
	K	SLVVTDYSTGK	E		1169	74	5		
	K	SNFYIMYGK	T		1122	42	1		
	K	WFTAEDNPGK	I		1164	48	3		
	R	YQQAQEDFAK	L		1227	70	6		
Utr2 / CAGL0C02211g	K	DFTQDSIMISDK	D	CaZy GH16	1399	72	1		
	K	FDDYSSK	V		860	37	1		
	K	NSGGSVLSTTR	Y		1078	97	5		
Scw4 / CAGL0G00308g	K	DASAVASDLAALSQYPTIR	L	CaZy GH17	1948	37	1		
	K	GQTLGVAVPSK	Q		1056	71	1		
	K	KPDTQVGGAGVK	G		1156	62	1		
Gas1 / CAGL0G00286g	A	DDLPAIEIK	G	CaZy GH72	1013			48	1
	R	KSPSWDLEIFDR	Y		1492	63	1		
	K	SPSWDLEIFDR	Y		1364	62	2		
Gas2 / CAGL0M13849g	R	DSPSWDLELYER	Y	CaZy GH72	1509	65	1		
Gas4 / CAGL0F03883g	R	DAFVFQQLGINTIR	V	CaZy GH72	1621	97	1		
	R	TFDEVSEGLYGGLK	A		1514	75	1		
	R	VYTLNPDINHDK	C		1428	67	1		
Gas5 / CAGL0F01287g	K	GNAFFDSSSNER	F	CaZy GH72	1330	73	1		
Cwp1.2 / CAGL0F07579g	K	SGGSFAGTVTDAGK	L	Cwp1 family	1254	107	72		
Cwp1.1 / CAGL0F07601g and Cwp1.2	L	AIHSGSPVQNTPV(D/N)S(E/Q)NGALVLK[d]	T	Cwp1 family non-unique peptides	2331			67	3
Cwp1.1 and 1.2	L	AIHSGSPVQNTPV(D/N)S(E/Q)NGALVLK Deamidated (NQ)	T		2332			44	1
Cwp1.1 and 1.2	F	AIPSGSEYK	F		951			42	1
Cwp1.1 and 1.2	F	ALSGSHLTYK	G		1076			71	6
Cwp1.1 and 1.2	Y	AVVNSDGSIK	T		989			63	1
Cwp1.1 and 1.2	N-term[c]	DSQAFGLLAIHSGSPVQNTPV(D/N)S(E/Q)NGALVLK	S or T		3163			50	1
Cwp1.1 and 1.2	N-term	DSQAFGLLAIHSGSPVQNTPV(D/N)S(E/Q)NGALVLK Deamidated (NQ)	S or T		3164			61	3
Cwp1.1 and 1.2	N-term	DSQAFGLLAIHSGSPVQNTPV(D/N)S(E/Q)NGALVLK 2 Deamidated (NQ	S or T		3165			50	1
Cwp1.1 and 1.2	K	FSDNTYAVVNSDGSIK	T		1716	136	121		
Cwp1.1 and 1.2	K	FSDNTYAVVNSDGSIK Deamidated (NQ)	T		1717			109	2
Cwp1.1 and 1.2	K	FSTAQGTGAIDIVISPR	S		1732	133	123		
Cwp1.1 and 1.2	K	FSTAQGTGAIDIVISPR Deamidated (NQ)	S		1733			75	3
Cwp1.1 and 1.2	F	GLLAIHSGSPVQNTPV(D/N)S(E/Q)NGALVLK	S or T		2614			61	2
Cwp1.1 and 1.2	F	GLLAIHSGSPVQNTPV(D/N)S(E/Q)NGALVLK Deamidated (NQ)	S or T		2615			72	5
Cwp1.1 and 1.2	K	GNSGFFAIPSGSEYK	F		1560	69	33		
Cwp1.1 and 1.2	K	GNSGFFAIPSGSEYK Deamidated (NQ)	F		1561			55	5
Cwp1.1 and 1.2	G	GSFAGTVTDAGK	L		1110			52	2
Cwp1.1 and 1.2	Q	GTGAIDIVISPR	S		1198			64	1
Cwp1.1 and 1.2	A	IHSGSPVQNTPV(D/N)S(E/Q)NGALVLK Deamidated (NQ)	S or T		2261			45	1
Cwp1.1 and 1.2	L	LAIHSGSPVQNTPV(D/N)S(E/Q)NGALVLK	S or T		2444			49	1
Cwp1.1 and 1.2	L	LAIHSGSPVQNTPV(D/N)S(E/Q)NGALVLK Deamidated (NQ)	S or T		2445			60	3
Cwp1.1 and 1.2	N	SGFFAIPSGSEYK	F		1389			86	6
Cwp1.1 and 1.2	L	SGSHLTYK	G		891			50	6
Cwp1.1 and 1.2	H	SGSPVQNTPV(D/N)S(E/Q)NGALVLK	S or T		2010			88	3
Cwp1.1 and 1.2	H	SGSPVQNTPV(D/N)S(E/Q)NGALVLK Deamidated (NQ)	S or T		2011			76	3
Cwp1.1 and 1.2	D	SQAFGLLAIHSGSPVQNTPV(D/N)S(E/Q)NGALVLK Deamidated (NQ)	S or T		3049			71	1
Cwp1.1 and 1.2	F	STAQGTGAIDIVISPR	S		1585			59	1
Cwp1.1 and 1.2	K	TGSESEGTSGFAL	S		1242			75	4
Cwp1.1 and 1.2	K	TGSESEGTSGFALSGSH	L		1610			108	4
Cwp1.1 and 1.2	K	TGSESEGTSGFALSGSHL	T		1723			85	1
Cwp1.1 and 1.2	K	TGSESEGTSGFALSGSHLT	Y		1824			70	2
Cwp1.1 and 1.2	K	TGSESEGTSGFALSGSHLTYK	G		2115	130	37		
Cwp1.1 and 1.2	N	TPVDSQNGALVLK	S or T		1341			117	2
Cwp1.1 and 1.2	N	TPVDSQNGALVLK Deamidated (NQ)	S or T		1342			53	1
Ecm33 / CAGL0M01826g	K	EVNVLNINNNR	Y	Ecm33 family	1298	66	3		
	K	GAIQGDSFVCK	N		1181	76	3		
Pst1 / CAGL0E04620g	K	GGIQGDNFVCK	N	Ecm33 family	1194	48	2		
Plb1 / CAGL0J11770g	R	ALSYQFFPTLYR	G	Plb phospholipase family	1505	64	2		
	K	DLSEDYDDIAVYAPNPFR	D		2099	49	1		
	R	GGVAYLWSDLR	E		1236	68	1		
Plb2 /	R	DNEIFMNGEMPMPISVADGR Deamidated (NQ)	Y	Plb phospholipase	2223			68	1
	F	EIGSWDPSLNAFSDIK	Y		1778			82	2
	R	NAADGLSPAEK	E		1072	56	3		

CAGL0J11748g	R	NAFEATTR	N	family	909	54	3		
	R	WTAIGSQVQGK	R		1174	75	1		
	K	YLGTQVTDGKPETER	C		1693	55	1		
Pir2 / CAGL0I06182g	K	APNDPVGAVSCK	V	Pir family	1214	78	14		
	N	FYNLYDQHIGSQCTPVHLSAIDLIK Deamidated (NQ)	C		2932			45	1
	P	NDPVGAVSCK	V		1046			60	1
	K	TAVSQIGDGQIQATK Deamidated (NQ)	T		1517			60	1
Pir3 / CAGL0M08492g	K	DPKDPVGAVSCK	V	Pir family	1272	43	1		
	N	FYNLYDQSIGAQCHPVHLSAIDLVK	C		2887			54	1
	N	FYNLYDQSIGAQCHPVHLSAIDLVK Deamidated (NQ)	C		2888			47	1
	K	GGILTDEK	G		831	49	17		
	H	LSAIDLVK	C		858			41	1
	L	SGNFYNLYDQSIGAQCHPVHLSAIDLVK Deamidated (NQ)	C		3147			50	1
	K	VDGTLQMNLK	G		1118	85	13		
	K	VDGTLQMNLK Oxidation (M)	G		1134	61	7		
Pir4 / CAGL0I06160g	K	IAEQCSPINLEVVSLVDC	-	Pir family	2045	118	2		
	C	LSGNFYNLYDQK	I		1461			69	2
	K	NDGTLQLTLK	G		1102	78	9		
	K	NDGTLQLTLK Deamidated (NQ)	G		1103			50	1
	R	QFQFDGPPPQAGAIYAGGW	S		2006			113	1
	L	SGNFYNLYDQK	I		1348			40	2
	R	VGSIVSNR	Q		830	58	13		
Pir1/CAGL0I06204g and Pir2	Q	CTPVHLSAIDLIK	C	Pir family non-unique peptides	1466			46	1
Pir1 and 2	R	QFQFDGPPPQAGAIF	A		1619			68	2
Pir1 and 2	R	QFQFDGPPPQAGAIFAA	G		1761			44	1
Pir1, 2 and 3	R	IGSIVANR	Q		828	65	16		
Pir1, Pir2 (and Pir3_deamidated)	K	VDGTLEMNLK	G		1119	72	3		
Pir1, 2, 3, 4 and 5	R	QFQFDGPPPQAG	A		1288			47	1
Pir1, 2, 3, 4 and 5	R	QFQFDGPPPQAGA	I		1358			52	1
Pir2 and 3	K	DPVGAVSCK	V		931	59	11		
Pir3 and 5	R	QFQFDGPPPQAGAIYAA	G		1777			52	1
Pir3 and 5	R	QFQFDGPPPQAGAIYAAGW	S		2020			96	2
Pir3, 4 and 5	R	QFQFDGPPPQAGAIY	A		1635			71	2
Tir1 / CAGL0F01463g	K	GIEALLPK	L	Srp1/Tip1 family	840	62	12		
	K	SYTSLYPSVDFK	G		1406	50	1		
Tir2 / CAGL0F01485g	M	QLAELNAVMEDLK	T	Srp1/Tip1 family	1473			77	2
	M	QLAELNAVMEDLK Oxidation (M)	T		1489			73	1
	N-term	SNPTPMQLAELNAVMEDLK	T		2100			112	2
	N-term	SNPTPMQLAELNAVMEDLK Oxidation (M)	T		2116			49	1
	K	TNLQDYMNLAMDPN	S		1639			72	1
Ssr1 / CAGL0H06413g	N	GNSDAAYTAFK	N	Unknown function	1144			86	1
	K	NCLDSICPNGNSDAAYTAFK	N		2217	97	1		
	K	NCLDSICPNGNSDAAYTAFK Deamidated (NQ)	N		2218			141	5
	K	NCLDSICPNGNSDAAYTAFK 2 Deamidated (NQ)	N		2219			114	2
	K	QSSQCHTLNQVGCFCENENTAIK	N		2724	68	4		
	N	QVGCFCENENTAIK	N		1669			64	3
	A	TPPACLLACVAQVSK	Q		1614			103	4

[a]Indicated for each peptide is the Mascot score and the number of times the peptide was selected for MS/MS fragmentation.

[b]Blue color indicates semitryptic (ST) peptides.

[c]N-term of mature protein as predicted by SignalP.

[d]Mascot assignment of semitryptic peptides containing the sequence tags TPVDSQNGALVLK (Cwp1.1) or TPVNSENGALVLK (Cwp1.2) suggests that both proteins are present but is not unambiguous, therefore we have listed these peptides as "Cwp1.1 and 1.2".

SUPPLEMENTARY TABLE S2. Oligonucleotides used in this study.

Name of primer[a]	Sequence 5´-3´[b]	Function
AWP14-KpnI-F	CACAGGTACCGAGCTGGATTTCATGAGC	Cloning *AWP14* upstream flank.
AWP14-XhoI-R	CACACTCGAGCATGAACAAATGAAATTAGTAGG	
AWP14-NotI-F	CACAGCGGCCGCATTCCAGAACAGATGATGG	Cloning *AWP14* downstream flank.
AWP14-SacI-R	CACAGAGCTCGTATCCACCGTATTGATAAC	
pSFS1-5'-R	ATTTGAAGTTTTACTTGGC	Checking genomic integration of the cassette together with *AWP14* external primers.
pSFS1-3'-F	ACAATCAAAGGTGGTCCTGC	
AWP14-ext-F	ATGTCCCAACCAGAGAG	Checking integration and excision of the cassette and deletion of *AWP14*.
AWP14-ext-R	GGTTCGATGATAGGTATG	
AWP14-int_F	GACGTATGAAAACCAAAAAATTG	

[a]*F, forward; R, reverse.*
[b]*Restriction enzyme sites used for cloning are underlined.*
