# Supplementary figures and images for: Characterization of Awp14, A Novel Cluster III Adhesin Identified in a High Biofilm-Forming Candida glabrata Isolate
Source: Front Cell Infect Microbiol. 2021 Nov 15;11:790465. doi: 10.3389/fcimb.2021.790465 (PMC8634165; doi:10.3389/fcimb.2021.790465)

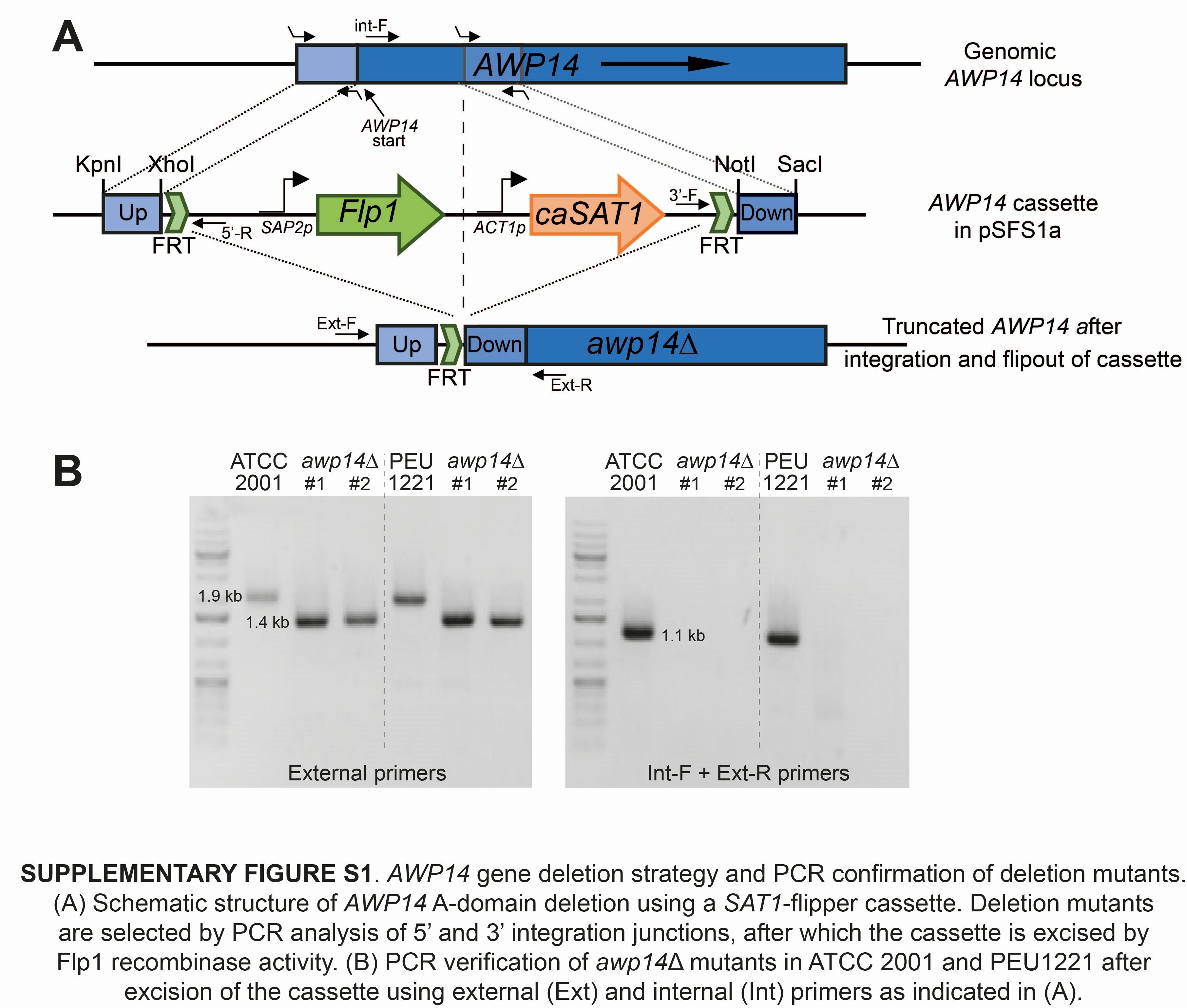

Supplement: Supplementary file 2 [file Image_1.jpeg]
